# Parents’ intention to vaccinate their child for COVID-19: A mixed-methods study (CoVAccS–wave 3)

**DOI:** 10.1371/journal.pone.0279285

**Published:** 2022-12-27

**Authors:** Louise E. Smith, Susan M. Sherman, Julius Sim, Richard Amlôt, Megan Cutts, Hannah Dasch, Nick Sevdalis, G. James Rubin

**Affiliations:** 1 King’s College London, Institute of Psychiatry, Psychology and Neuroscience, London, United Kingdom; 2 NIHR Health Protection Research Unit in Emergency Preparedness and Response, England; 3 Keele University, School of Psychology, Newcastle, United Kingdom; 4 Keele University, School of Medicine, Newcastle, United Kingdom; 5 UK Health Security Agency, Behavioural Science and Insights Unit, England; 6 King’s College London, Centre for Implementation Science, London, United Kingdom; Institute of Psychiatry, Psychology & Neuroscience, King’s College London, UNITED KINGDOM

## Abstract

**Aim:**

To investigate UK parents’ vaccination intention at a time when COVID-19 vaccination was available to some children.

**Methods:**

Data reported are from the second wave of a prospective cohort study. We conducted a mixed-methods study using an online survey of 270 UK parents (conducted 4–15 October 2021). At this time, vaccination was available to 16- and 17-year-olds and had become available to 12- to 15-year-olds two weeks prior. We asked participants whose child had not yet been vaccinated how likely they were to vaccinate their child for COVID-19. Linear regression analyses were used to investigate factors associated with intention (quantitative component). Parents were also asked for their main reasons behind vaccination intention. Open-ended responses were analysed using content analysis (qualitative component).

**Results:**

Parental vaccination intention was mixed (likely: 39.3%, 95% CI 32.8%, 45.7%; uncertain: 33.9%, 95% CI 27.7%, 40.2%; unlikely: 26.8%, 95% CI 20.9%, 32.6%). Intention was associated with: parental COVID-19 vaccination status; greater perceived necessity and social norms regarding COVID-19 vaccination; greater COVID-19 threat appraisal; and lower vaccine safety and novelty concerns. In those who intended to vaccinate their child, the main reasons for doing so were to protect the child and others. In those who did not intend to vaccinate their child, the main reason was safety concerns.

**Conclusions:**

Parent COVID-19 vaccination status and psychological factors explained a large percentage of the variance in vaccination intention for one’s child. Further study is needed to see whether parents’ intention to vaccinate their child is affected by fluctuating infection rates, more children being vaccinated, and the UK’s reliance on vaccination as a strategy to live with COVID-19.

## Introduction

Vaccination has been one of the cornerstones of the public health response to COVID-19. However, there has been some debate over the need to vaccinate children due to the relatively lower severity of infection [1]. In the UK, vaccination has been available for those aged 16 years and above since December 2020, but was subsequently extended to younger age groups (see Box 1).

**Box 1. Timeline of recommendations for COVID-19 vaccination in children and adolescents in England**

**Table:** 

2 December 2020. MHRA gives approval for Pfizer/BioNTech vaccine in people aged 16 years and above [2] 4 June 2021. MHRA gives approval for Pfizer/BioNTech vaccine in 12- to 15-year-olds [3] 19 July 2021. JCVI advises that children and young people aged 12 years and above with specific underlying health conditions that put them at serious risk of COVID-19 be offered COVID-19 vaccination (2 doses, with an 8-week gap) [4] 4 August 2021. JCVI advises that all adolescents aged 16 to 17 years receive first dose of Pfizer/BioNTech vaccine [5] 17 August 2021. MHRA gives approval for Moderna vaccine for 12- to 17-year-olds [6] 2 September 2021. Half of 16- and 17-year-olds had received first COVID-19 vaccine [7] 3 September 2021. JCVI release a report on vaccinating healthy 12- to 15-year-olds saying that “the health benefits from COVID-19 vaccination to healthy children aged 12 to 15 years are marginally greater than the potential harms … the margin of benefit is considered too small to support universal COVID-19 vaccination for this age group at this time” [8] 13 September 2021. Children aged 12- to 15-years old in England will be offered one dose of Pfizer/BioNTech vaccine, following advice from the four UK Chief Medical Officers [9] 14 September 2021. JCVI recommends a third dose (booster) for 16- to 49-year-olds with underlying health conditions [10] 20 September 2021. NHS starts vaccine rollout in schools to children aged 12- to 15-year-olds [11] 15 November 2021. JCVI recommends a second vaccine dose for all 16- to 17-year-olds (12 weeks or more after first dose) [12] 25 November 2021. European Medicines Agency recommends approval for Pfizer/BioNTech vaccine for 5- to 11-year-olds [13] 29 November 2021. JCVI advises that all 12- to 15-year-olds should be offered a second dose of Pfizer-BioNTech vaccine [14] 13 December 2021. NHS COVID-19 vaccine pass for international travel introduced for 12- to 15-year-olds [15] 22 December 2021. MHRA gives approval for Pfizer vaccine for 5- to 11-year-olds [16] 22 December 2021. JCVI recommends that children aged 5 to 11 years in a clinical risk group be offered COVID-19 vaccination (2 doses, with an 8-week gap). JCVI recommends that 16- to 17-year-olds and 12- to 15-year-olds in a clinical risk group or who are a household contact of someone who is immunosuppressed be offered a booster dose of the Pfizer-BioNTech vaccine no sooner than 3 months after they complete their primary vaccination course [17] 16 February 2022. JCVI advises a “non-urgent offer” of vaccination to all children aged 5 to 11 years (two doses of Pfizer-BioNTech vaccine, at least 12 weeks apart) [18]

Abbreviations: JCVI = Joint Committee on Vaccination and Immunisation, MHRA = Medicines and Healthcare products Regulatory Agency, NHS = National Health Service, UK = United Kingdom, UKHSA = UK Health Security Agency.

Multiple factors are important in parents’ vaccine decision making for their children. Previous research indicates that uptake of child vaccination is associated with: perceived vaccine safety, generally positive vaccine attitudes, perceived susceptibility of the child to infection and greater normative beliefs for vaccination [19, 20]. Findings from two systematic reviews and meta-analyses investigating parents’ COVID-19 vaccination intention for their children (searching data published up to December 2021) indicate that intention was associated with psychological factors including greater perceived risk of COVID-19, more positive vaccine attitudes, and greater perceived safety of COVID-19 vaccination [21, 22]. Novelty and a perceived lack of evidence about the effectiveness of child vaccination were associated with decreased parental intention [21]. Evidence for associations with parent and child sociodemographic characteristics was mixed, but parental COVID-19 vaccine uptake (or intention) was consistently associated with intention to vaccinate one’s child [21, 22].

Different countries have employed different COVID-19 vaccine strategies. A review of European vaccine laws conducted just before the pandemic found that there was no common “best approach” and that the context in individual countries needed to be considered when making recommendations [23]. To the best of our knowledge, only three studies have investigated parental intentions to vaccinate their child for COVID-19 in a UK sample, all of which were conducted before adult and child COVID-19 vaccination was approved in the UK (Box 1): data collection periods April to May 2020 [24], August to October 2020 [25], and October to November 2020 [26]. At this time, there was considerable uncertainty about whether to vaccinate children [27]. We set out to investigate parental vaccination intentions at a time when there was relatively more clarity about the COVID-19 vaccine rollout for children in the UK. These results may help inform communications about child COVID-19 vaccination.

This study aimed to investigate the following questions:

1. What were parents living in the UK’s intention to vaccinate their children against COVID-19 at a time when vaccination was available for some children?
2. What were the main reasons behind parents’ vaccination intentions?
3. Which factors (personal, clinical, psychological) were associated with vaccination intention?

## Methods

This study reports data from the COVID-19 vaccination acceptability study (CoVAccS). Methods have been reported in more detail elsewhere [28].

### Design

This formed part of a larger prospective cohort study, in which participants completed a survey online on 13–15 January 2021 (T1) and 4–15 October 2021 (T2). The wider study investigated uptake of adult vaccination [28] and factors affecting perception of vaccine side effects in those vaccinated [29]. For this study, we used personal and clinical characteristics reported at T1, and measured parental vaccination intention, reasons for vaccination intentions, and psychological factors at T2. We used mixed methods (qualitative analysis of reasons behind vaccination intentions, quantitative analysis of vaccination intentions and associated factors) to analyse the data. Qualitative and quantitative results were integrated to draw inferences and conclusions about parental intentions to vaccinate their child for COVID-19 [30].

### Participants

To be eligible for this study, participants had to be aged 18 years or over, live in the UK and have not completed our previous study (CoVAccS 1), due to similarities in survey materials [31]. 1500 participants completed the first wave of data collection (T1), with 1148 completing the second wave (response rate 76.5%; T2). For this study, we included participants if they indicated that they were the parent or legal guardian of a dependent child aged 17 years or younger (*n* = 270).

### Measures

Full survey materials are available online [32]. Bespoke measures, based on theories of health behaviour (e.g. Protection Motivation Theory [33], the Health Belief Model [34]) and previous literature [35–38], were used in this study. Unless otherwise specified, all items were measured at T2.

Parents were asked to complete the survey with one of their children in mind (index child). We asked parents to think about their child who had most recently had a birthday.

#### Personal and clinical characteristics

At T1, all participants were asked to provide information about their sex, age, ethnicity, religion, highest qualification, employment status, total household income, and region where they live. At T2, we asked whether they had a chronic illness and recoded people as being “at risk” of COVID-19 based on NHS guidance [39].

Parents provided the index child’s sex, age, whether this was their first child, whether the child had previously had COVID-19, and presence of a chronic illness (recoded to “at risk” of COVID-19 based on NHS guidance [39]).

#### Psychological factors

Parents were asked to respond to nine statements about COVID-19 and vaccination with reference to their child on an 11-point scale from “strongly disagree (0)” to “strongly agree (10)”. Items asked about perceived risk of COVID-19 to the child, worry about the child catching COVID-19, perceived susceptibility to, and severity of, COVID-19 for the child, anticipated regret about the child catching COVID-19 if they had not been vaccinated, belief that the vaccine could give the child COVID-19, regretting vaccination if the child were to experience side effects, worry about the safety of COVID-19 vaccination for children, and perceived social norms about child vaccination (operationalised as “most other children my child’s age will get the coronavirus vaccination”).

Parents were also asked about their agreement that vaccination is generally a good thing.

#### Vaccination uptake

Parents were asked if their child had been vaccinated against COVID-19. Response options were: “yes, they’ve had one dose”, “yes, they’ve had two doses”, “no”, “don’t know”, and “prefer not to say”.

Parents who indicated that they had not yet vaccinated their child were asked how likely it was that their child would have a COVID-19 vaccine eventually and for the main reason why their child was “likely or unlikely to have a coronavirus vaccination”.

### Ethics

We obtained ethical approval for this study from Keele University’s Research Ethics Committee (reference: PS-200129). Consent was given by participants before being able to complete each of the T1 and T2 survey materials. No minors were included in this study.

### Analysis

Parents who reported that their child had been vaccinated were excluded from analyses of vaccination intention.

We categorized parents as being very likely to vaccinate their child if they scored 8 to 10 on the 0–10 point numerical response scale, very unlikely to vaccinate their child if they scored 0 to 2, and uncertain about vaccinating their child if they scored 3 to 7. These conservative *a priori* cut-offs were chosen as they reflect the extreme three points of the scale, avoiding the chance of and false positives, and were used in our previous work [31, 40]. To investigate whether parents’ vaccine intentions differed by index child age (0 to 11 years, 12 to 15 years, 16 to 17 years), we used a one-way ANOVA.

To investigate the main reasons behind parents’ intention to vaccinate their child for COVID-19, we qualitatively analysed open-ended text responses using content analysis. First, two authors (MC and HD) identified emerging codes from the data [41]. A coding framework from analyses of previous CoVAccS work was used as support [40]. Statements were jointly coded, with any discrepancies being resolved through discussion to give a final set of codes. Data analysis of responses about parental vaccination intention was carried out at the same time as analyses of reasons for future own COVID-19 vaccination intentions detailed in Smith et al. [28]. This qualitative analysis was manual; no software was used.

We conducted a principal components analysis to identify underlying dimensions of psychological items. As components were thought likely to be correlated, we used oblique (direct oblimin) rotation. All psychological factor items pertaining to parents’ beliefs about COVID-19 illness and vaccination in their child were included in the analysis.

Factors associated with parents’ intention to vaccinate one’s child were investigated using a linear regression analysis (*n* = 224), using the full eleven-point response scale as the outcome measure. Variables were entered into the regression model in blocks; the order was selected *a priori* based on theoretical relevance (block 1: parent socio-demographic characteristics; block 2: child socio-demographic characteristics; block 3: general vaccination beliefs and attitudes; block 4: beliefs and attitudes about COVID-19 and vaccination). To control the rate of Type 1 errors in the regression analysis, we set statistical significance at *p*≤.01 and therefore calculated 99% confidence intervals (CIs) for regression coefficients.

Quantitative analyses were conducted using SPSS 27 [42].

### Power

We conducted a post-hoc power analysis based on linear regression analyses. With an achieved sample size of 220, a 1% two-tailed significance level, and testing 16 predictors, we had 88.8% power to detect medium effect sizes (*f*^2^ = 0.15).

## Results

### Participant characteristics

The 270 participants included in the study were mostly female (54.8%, *n* = 148/270), white (85.6%, *n* = 231/270), with a mean age of 42.1 years (SD = 9.3; Table 1). Most participants lived in the South East, followed by the West Midlands, and London.

**Table 1:** Participant characteristics. Data are percentages and frequencies, except for age: mean (SD). Data are for all parents regardless of the vaccination status of their child (total *n* = 270).

	Personal and clinical characteristic	Level	%	*n*
**Parent**	**Sex**	Female	54.8	148
		Male	45.2	122
	**Age**	Years, mean (SD)	42.1 (9.3)	270
	**Ethnicity**	White	85.6	231
		Black and minority ethnic	14.4	39
	**Religion**	No religion	54.1	146
		Christian	37.8	102
		Other religion	7.4	20
		Prefer not to say	0.7	2
	**Highest qualification**	Degree equivalent or higher †	57.8	156
		Other or no qualifications	41.9	113
		Prefer not to say	0.4	1
	**Employment status**	Full-time	58.5	158
		Part-time	20.7	56
		Not working/other	20.7	56
	**Total household income**	£30,000 or over	72.2	195
		Up to £29,999	21.9	59
		Don’t know/prefer not to say	5.9	16
	**Region where respondent lives**	East Midlands	7.4	20
		East of England	7.8	21
		London	11.1	30
		North East	3.0	8
		North West	9.3	25
		Northern Ireland	3.0	8
		Scotland	8.9	24
		South East	17.8	48
		South West	7.8	21
		Wales	3.0	8
		West Midlands	11.5	31
		Yorkshire and the Humber	9.3	25
		Prefer not to say	0.4	1
	**At risk**	No	86.3	233
		Yes	13.7	37
**Child**	**Sex**	Female	43.3	117
		Male	55.2	149
		Other/prefer not to say	1.5	4
	**Age**	Years, mean (SD)	9.5 (5.4)	270
	**First child**	No	44.1	119
		Yes	54.8	148
		Prefer not to say	1.1	3
	**At risk**	No	97.0	262
		Yes	3.0	8
	**Previously had COVID-19**	Do not know or think not	75.2	203
		Think or know yes	24.8	67

† Undergraduate (e.g. BA, BSc) or postgraduate (e.g. MA, MSc, PhD) degree or other technical, professional or higher qualification.

### Vaccine uptake

Three percent of parents (*n* = 8, 95% CI 1.5% to 5.7%) reported that their child had had two doses of the vaccine, with 14.1% (*n* = 38; 95% CI 10.4% to 18.7%) reporting that their child had had one dose of the vaccine, and 83.0% (*n* = 224, 95% CI 78.0% to 87.0%) reporting that their child had not received the vaccine.

### Vaccine intention

**Fig 1:**
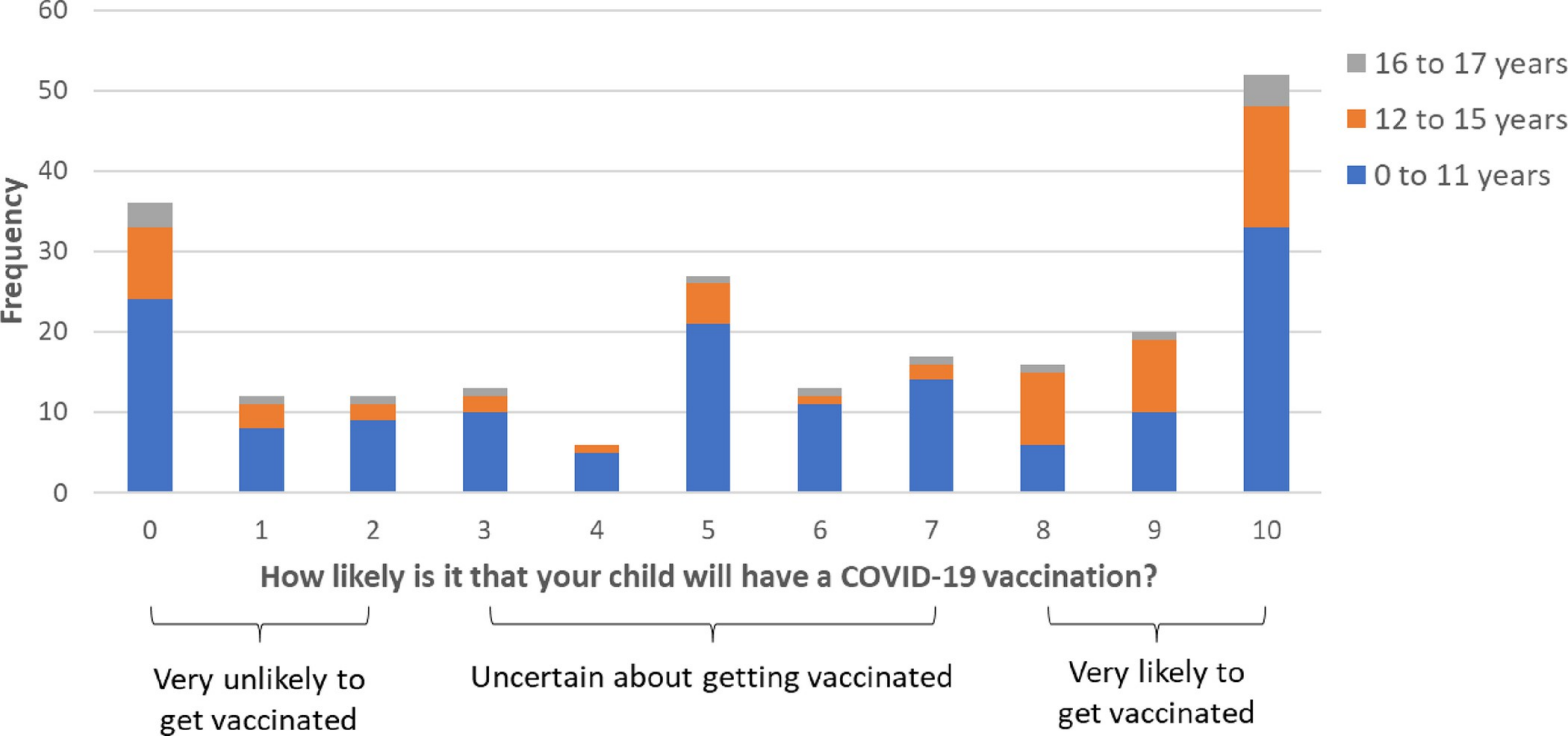
Perceived likelihood of child having a vaccination (0 = “extremely unlikely” to 10 = “extremely likely”) by child age, with cut-off points used to categorize participants’ vaccination intention.

Parents who had not yet vaccinated their child were split in their intention to vaccinate their child (Fig 1). Of parents of children of all ages, 39.3% (95% CI 33.1% to 45.8%, *n* = 88/224) were likely to vaccinate their child, 33.9% (95% CI 28.0% to 40.4%,; *n* = 76/224) were uncertain, and 26.8% (95% CI 21.4% to 32.9%, *n* = 60/224) were unlikely to vaccinate their child. The mean (SD) parental intention score was 5.62 (3.68), and was similar in relation to children in different age bands: 5.38 (3.59) for those aged 0–11; 6.29 (3.77) for those aged 12–15; 5.40 (4.07) for those aged 16–17.

### Reasons behind intention

The main reasons behind vaccination intention in parents who were likely to vaccinate their child in qualitative analyses were to protect the child, to protect others, and because the child had chosen to (Table 2). The main reasons behind intention in parents who were unlikely to vaccinate their child were safety concerns, feeling that the threat to the child of COVID-19 was low, and that there was no personal need for the child to be vaccinated.

**Table 2:** Thematic categorization of codes generated by content analysis of reasons for or against vaccinating one’s child, by vaccination intention. Themes are presented in descending order of overall frequency.

Theme	Codes	Frequency		
		Vaccination intention		
		Unlikely	Uncertain	Likely
**Safety concerns [total = 94]**	Child too young	18	16	5
	Lack of research on the vaccine	11	6	1
	Concerns about vaccine side effects and safety	8	8	
	Concerns about long-term vaccine side effects	6	3	
	Vaccine side effects outweigh benefits	3	1	
	Vaccine is more risky than the virus	2	1	
	Vaccines use experimental mRNA/novel technology	1		
	Have heard negative stories about the vaccine	1		
	Fertility concerns		1	
	Concerns about quick development of the vaccine		1	
	Anxiety about the vaccine		1	
**To protect child [total = 63]**	Protecting the child		8	40
	Vaccine reduces disease severity/fatality		2	4
	Virus is more risky than the vaccine		2	1
	Would regret not vaccinating if child got severely ill		1	1
	Concerns about long-term adverse effects of virus		1	1
	Vulnerable due to underlying health condition		1	
	To speed up medical attention			1
**To protect others [total = 29]**	Protecting the wider community		3	7
	Protecting family		2	4
	To reduce the spread		1	9
	Protecting the vulnerable		1	1
	To gain herd immunity			1
**Child choice [total = 27]**	Letting child decide	2	4	2
	Child does not want the vaccine	3	1	
	Child too young to make informed choice	1	1	
	Child decided to get vaccinated			9
	Child thinks it is the best thing to do/sees benefits of vaccination			4
**Low threat appraisal [total = 26]**	Risk for children is low	12	12	1
	Limited contact with others		1	
**Future intention [total = 22]**	Undecided		10	
	Will have when available for age group		3	4
	Not offered yet		1	1
	Child will have the vaccine			3
**Information [total = 11]**	Lack of knowledge about vaccine	1	5	
	Conflicting information	1	1	
	Differing opinions among caregivers		1	
	Social influence		1	
	Following medical advice	1		
**Positive vaccine views [total = 11]**	Parent in favour of vaccine		2	2
	Pro-vaccine in general		1	4
	Sensible thing to do			2
**No personal need [total = 10]**	Vaccine is unnecessary	6		
	Child has no personal need for the vaccine	2	1	
	Only people at high-risk need the vaccine	1		
**To end pandemic and restrictions [total = 8]**	To overcome the pandemic		1	2
	To prevent future mutations			2
	Prevent missing school days			3
**Natural immunity [total = 7]**	Likely already had COVID	2		1
	Prefer natural immunity/think natural immunity is sufficient	3		
	Antibodies provide protection via breastfeeding	1		
**Trust in science [total = 7]**	Would vaccinate child if evidence based		4	1
	Trust in the vaccine			1
	Trust in science			1
**Move about freely [total = 6]**	For personal freedom		2	1
	Wanting to travel		1	1
	To gain an immunity passport			1
**Lack of trust in science [total = 5]**	Lack of trust in the vaccine	3	1	
	Lack of trust in science		1	
**Social / civic duty [total = 4]**	Irresponsible not to			1
	Vaccine is a civic duty/social responsibility			1
	Mass vaccination needed			1
	Vital to have the vaccine			1
**Vaccine mode [total = 4]**	Fear of needles	1		2
	Depending on administration method (no injections)		1	
**Vaccine not effective [total = 2]**	Concerns regarding the effectiveness of vaccine	1		
	Vaccine does not stop COVID transmission		1	
**High threat appraisal [total = 2]**	Higher risk for adolescents			1
	High number of cases in age group			1

### Principal components analysis

A scree plot identified that psychological items loaded onto three main components. The item “The coronavirus vaccination could give my child coronavirus” did not load on to any component. We therefore re-ran the principal components analysis excluding this variable. Table 3 shows item loadings on to components.

**Table 3:** Loadings of items measuring psychological factors onto components identified (only loadings over ± .400 are shown).

Component 1		Component 2		Component 3	
Perceived necessity and social norms of child COVID-19 vaccination		COVID-19 threat appraisal		Vaccine safety and novelty concerns	
Without a coronavirus vaccination, my child is likely to catch coronavirus	.772	To what extent do you think coronavirus poses a risk to your child?	.855	I might regret my child getting the coronavirus vaccination if they later experienced side effects from it	.934
If my child doesn’t get the coronavirus vaccination and ends up getting coronavirus, I will regret them not getting the vaccination	.586	I am worried about my child catching coronavirus	.723		
Most other children my child’s age will get the coronavirus vaccination	.806	I believe that coronavirus would be a mild illness for my child	–.832		
Coronavirus vaccination for children is too new for me to be confident about it	–.423			Coronavirus vaccination for children is too new for me to be confident about it	.685

Rotation method: oblimin with Kaiser normalization. Rotation converged in 7 iterations.

Excluding “The coronavirus vaccination could give my child coronavirus” item

Three components emerged from the principal components analysis on beliefs and attitudes about COVID-19 and vaccination, accounting for 72% of the variance in the original items (see S1 Checklist). One component related to illness beliefs and attitudes (‘COVID-19 threat appraisal’), and two components related to vaccination beliefs and attitudes (‘perceived necessity and social norms of child COVID-19 vaccination’ and ‘vaccine safety and novelty concerns’).

### Factors associated with parental vaccination intention

219 participants had complete data and were included in regression analyses. The overall regression model explained 66.9% of the variance, with parents’ beliefs and attitudes about COVID-19 and vaccination explaining the largest percentage of variance. Vaccination intention was associated with: being vaccinated oneself; greater perceived necessity and social norms regarding COVID-19 vaccination; greater COVID-19 threat appraisal; and lower vaccine safety and novelty concerns (Table 4).

**Table 4:** Results of the full linear regression model analysing associations with parental vaccination intention (adjusted *R*^2^ = 0.669). Parameter estimates relate to the full model containing all predictors. The unstandardized regression coefficients represent the change in parental vaccination intention for a one-unit increase in the predictor variable (or, for dummy variables, a shift from the reference category to the category concerned). The model was based on 219 study participants with complete data.

Predictor variable	Level	Standardized coefficient	Unstandardized coefficient	99% confidence interval	*p* value
**Block 1 –parent personal and clinical characteristics** ^**a**^					
**Sex (reference: female)**	Male	–0.036	–0.264	–1.171, 0.643	0.450
**Age (years)**		0.026	0.011	–0.050, 0.072	0.639
**Ethnicity (reference: white)**	Black and minority ethnic	0.097	1.061	–0.237, 2.359	0.035
**Religion (reference: none)**					
	Christian	0.003	0.025	–0.809, 0.858	0.938
	Other	–0.034	–0.538	–2.434, 1.357	0.461
**Qualifications (reference: other)**	Degree equivalent or higher	0.006	0.044	–0.812, 0.900	0.894
**Employment status (reference: not working/other)**					
	Part-time	0.076	0.693	–0.508, 1.894	0.135
	Full-time	0.026	0.195	–0.863, 1.254	0.632
**At risk–self (reference: no)**	Yes	–0.104	–1.158	–2.340, 0.025	0.012
**Vaccinated for COVID-19 –self (reference: no)**	Yes	0.157	1.695	0.336, 3.054	0.001*
**Block 2 –child personal and clinical characteristics** ^**b**^					
**Sex (reference: female)**	Male	–0.013	–0.096	–0.895, 0.703	0.755
**Age (years)**		–0.043	–0.032	–0.138, 0.073	0.428
**First child (reference: not)**	Yes	0.040	0.303	–0.552, 1.158	0.357
**Had covid before (reference: think not)**	Yes	0.067	0.600	–0.350, 1.550	0.102
**At risk–child (reference: no)**	Yes	–0.001	–0.033	–3.092, 3.025	0.977
**Block 3 –general vaccination beliefs and attitudes** ^**c**^					
**In general, vaccination is a good thing (0–10)**	0 = strongly disagree, 10 = strongly agree	0.125	0.246	–0.009, 0.500	0.013
**Block 4 –beliefs and attitudes about COVID-19 and vaccination** ^**d**^					
**Component: perceived necessity and social norms of child COVID-19 vaccination**		0.506	1.884	1.396, 2.372	<0.001*
**Component: COVID-19 threat appraisal**		0.141	0.521	0.089, 0.953	0.002*
**Component: vaccine safety and novelty concerns**		–0.277	–1.020	–1.430, –0.610	<0.001*

* *p*≤.01

^a^ variables in this block explained 29.2% of the variance

^b^ variables in this block explained a further 0.6% of the variance

^c^ variables in this block explained a further 7.1% of the variance

^d^ variables in this block explained a further 30.0% of the variance.

## Discussion

Of parents who had not yet vaccinated their children for COVID-19, intention to do so was mixed. Vaccination intention was associated with having received a COVID-19 vaccine oneself, perceiving COVID-19 vaccination as necessary, thinking that others would vaccinate their child, perceiving a greater threat of COVID-19, and thinking that the vaccine was safe. The most common reasons for intending to vaccinate were for the protection of the child and others, while the most common reason for intending not to vaccinate was safety concerns.

By the end of data collection, COVID-19 vaccination was available to all 16- and 17-year-olds and had recently become available to 12- to 15-year-olds in England (see S1 Checklist). Official NHS figures indicate that, at this time, 1,234,290 under-18s had had one COVID-19 vaccine dose, 251,825 had had two doses, and 1,117 had received a booster [43]. This equates to approximately 9% of the English population under 18 [44]. Uptake in our sample was slightly higher, with approximately 17% indicating that their child had been vaccinated.

Of those who had not yet vaccinated their child, parents’ vaccination intentions were mixed. Only 39% indicated they were likely to vaccinate their child (39%), lower than recent meta-analytic results, which found that approximately 60% were willing [21, 22]. It was also lower than other UK parental vaccination studies. One study of parents living in England with a child aged 18 months or younger found that 48% definitely would accept a COVID-19 vaccine for their child (data collected 19 April to 11 May 2020) [24]. UK data from a multi-country survey found that 64% of women who were pregnant or had one child aged 18 years or under were likely to vaccinate their child (data collected 28 October to 18 November 2020) [26]. The discrepancy between these findings and our results may be because of the increased debate surrounding child COVID-19 vaccination [1], and decreased perceived risk of COVID-19 between January and October 2021 [28], itself associated with vaccination intention [20].

Having had a COVID-19 vaccine oneself was strongly associated with parental vaccine intentions for their child, in line with systematic review results [21, 22]. This is the same pattern of results seen in previous pandemics, with intention to be vaccinated for pandemic influenza being associated with previous seasonal influenza vaccination during the 2009/2010 H1N1 influenza pandemic [45]. We found no other associations between parental vaccination intention for their child and parent or child sociodemographic characteristics. This is likely due to low power to detect smaller effects. Official UK figures show there are differences in uptake of vaccination in children aged 12 to 15 years by sociodemographic variables, with vaccine uptake being higher in children who are of Chinese and Indian ethnicities, live in less deprived areas, speak English as a first language, do not receive free school meals, and do not have special educational needs [46].

Beliefs and attitudes about COVID-19 and vaccination explained most of the variance in parents’ vaccination intentions. Intention was associated with greater perceived necessity of COVID-19 vaccination and social norms (believing that most other children will receive a COVID-19 vaccine), greater perceived risk of COVID-19, and greater perceived safety of vaccination. This is in line with other results found during the COVID-19 pandemic [21, 22], routine childhood vaccination [19, 20], and theories of uptake of health behaviours (e.g. the Protection Motivation Theory [33]). High case numbers in primary and secondary school age children, such as those seen in September to November 2021 and January 2022 [47], may also affect parents’ vaccination intention, through perceived susceptibility to infection. Factors associated with parental vaccination intention were similar to those associated with individuals’ own uptake of COVID-19 vaccination [28].

Parents who intended to vaccinate their child most commonly cited the protection of the child and others as the main reasons behind their intentions. A similar pattern of results has been found in children aged 12 to 18 years (UK school years 7 to 13) [48]. For those who did not intend to vaccinate their child, the main reasons were safety concerns and not perceiving COVID-19 to be a great threat to their child. This reflects findings from the Office for National Statistics, in which the main reasons behind parents being unlikely to agree to their child being vaccinated were worry about side effects and waiting to see how the vaccine worked [48].

### Implications of the findings for future practice and research

As the UK’s response to the pandemic shifts to “living with COVID-19,” this strategy is in part relying on adult vaccination as a means of reducing serious infection in the absence of other non-pharmaceutical interventions (testing, self-isolation, wearing a face covering, limits on social mixing) [49]. The risks and benefits of vaccination to children have been more balanced [17], leading to greater debate about whether children should be vaccinated [1]. Parents need to be able to make an informed decision as to whether they vaccinate their child. Since data collection, COVID-19 vaccination has been approved and recommended in younger age groups. Factors affecting parents’ decision to vaccinate their child are numerous and likely interlinked [19, 20]. While social norms for vaccination may increase with time as more children are vaccinated and perceptions of vaccine novelty may decrease, the landscape of the COVID-19 pandemic is constantly changing, with the risk of new variants and changes in restrictions. Official data indicate that up to 31 August 2022 (most recent data available at time of writing), 11.2% of 5- to 11-year-olds, 57.5% of 12- to 15-year-olds and 73.1% of 16- to 17-year-olds have received at least one COVID-19 vaccine [50]. Further research is needed to identify how fluctuating infection rates and decreased emphasis on COVID-19 in everyday life may affect parents’ perceived risk of their child being infected and, in turn, their vaccination intention and uptake by child age.

The most common reasons for intending to vaccinate one’s child for COVID-19 were to protect the child and others and because the child had chosen to be vaccinated. A study conducted in UK schoolchildren aged 9 to 18 years indicated that children who spent longer on social media, came from more deprived backgrounds, and smoked or vaped were less likely to intend to be vaccinated for COVID-19 [51]. Taken together, these results suggest that disseminating information about child vaccination on social media targeted to adolescents may increase vaccine uptake in this age group. As in previous research, the perception that the vaccine is safe and effective was associated with parent vaccination intention [19–22]. Communications should emphasize that child COVID-19 vaccination is safe and encourage parents to make an informed decision about the vaccine.

### Strengths and limitations

We measured self-reported intention to vaccinate one’s child when the vaccine was not yet available to children of most age groups. While the study was well powered to detect medium effect sizes, our sample size had limited power to detect smaller effect sizes. However, our regression model explained 67% of the variance in parents’ vaccination intention. This indicates good explanatory power for a study using these methods, with social science research and public opinion surveys typically giving low *R*^*2*^ values [52, 53]. This study was part of a prospective cohort study. Participants recruited into the study at T1 were broadly representative of UK adults based on age, sex and ethnicity [40]. Questions about child vaccination were only asked at T2, to those who completed the follow-up survey (response rate 76.5%) and who indicated that they were the parent or guardian of a child aged 17 years or under [28]. We cannot be sure that the sample included in this survey is representative of UK parents. Not all measures that we used have been validated. However, items were based on theories of health behaviour (e.g. Protection Motivation Theory [33], Health Belief Model [34]) and previous literature, including validated measures [35]. Participants were not geographically representative of the UK. Given that vaccine uptake has been found to differ by region in the UK [46], our results on intended uptake may not be representative of the general population. However, we have no reason to believe that factors associated with vaccination intention differ by region. We did not collect more detailed data on the region where participants lived (e.g., urban or rural areas, local authority) in an attempt to maintain respondent anonymity.

## Conclusion

It is important to understand parents’ COVID vaccination intentions for their child and factors associated with intention to help inform child vaccine communications. Parents’ COVID-19 vaccination intention for their child was mixed at a time when the vaccination was available for some children. Vaccination intention was associated with having been vaccinated for COVID-19 oneself, greater perceived necessity of, and social norms for, vaccination, greater perceived threat of COVID-19, and greater perceived safety of COVID-19 vaccination for children. Parents most commonly reported that they intended to vaccinate their child to protect the child and others, while the main reason behind not intending to vaccinate one’s child being due to safety concerns.

## Supporting information

S1 Checklist (DOC)
